# Supplementary material for: Loss of tumor suppressors promotes inflammatory tumor microenvironment and enhances LAG3+T cell mediated immune suppression
Source: Nat Commun. 2024 Jul 12;15:5873. doi: 10.1038/s41467-024-50262-8 (PMC11245525; doi:10.1038/s41467-024-50262-8)
Supplement: Supplementary file 1 — Supplementary Information [file 41467_2024_50262_MOESM1_ESM.pdf]

Supplementary Information

Loss of tumor suppressors promotes inflammatory tumor microenvironment and enhances LAG3+T cell mediated immune suppression

Sara Zahraeifard[1#], Zhiguang Xiao[1#], Jae Young So[1#], Abdul Ahad[1], Selina Montoya[1], Woo Yong Park[1], Trinadharao Sornapudi[1], Tiffany Andohkow[1], Abigail Read[1], Noemi Kedei[2], Vishal Koparde[3,4], Howard Yang,[1] Maxwell Lee,[1] Nathan Wong[3,4] Maggie Cam[3], Kun Wang[5], Eytan Ruppin[5], Ji Luo[1], Christine Hollander[1], Li Yang[1*]

[1]Laboratory of Cancer Biology and Genetics, Center for Cancer Research, National Cancer Institute, National Institutes of Health, Bethesda, MD 20892, USA

[2] Collaborative Protein Technology Resource, Center for Cancer Research, National Cancer Institute, National Institutes of Health, Bethesda, MD 20892, USA

[3]Collaborative Bioinformatics Resource, Center for Cancer Research, National Cancer Institute, National Institutes of Health, Bethesda, MD 20892, USA

[4]Advanced Biomedical Computational Sciences, Frederick National Laboratory for Cancer Research, Leidos Biomedical Research, Inc., Frederick, MD 21701, USA

[5] Cancer Data Science Laboratory, Center for Cancer Research, National Cancer Institute, National Institutes of Health, Bethesda, MD 20892, USA

# equal contribution

* corresponding author: yangl3@mail.nih.gov

S1

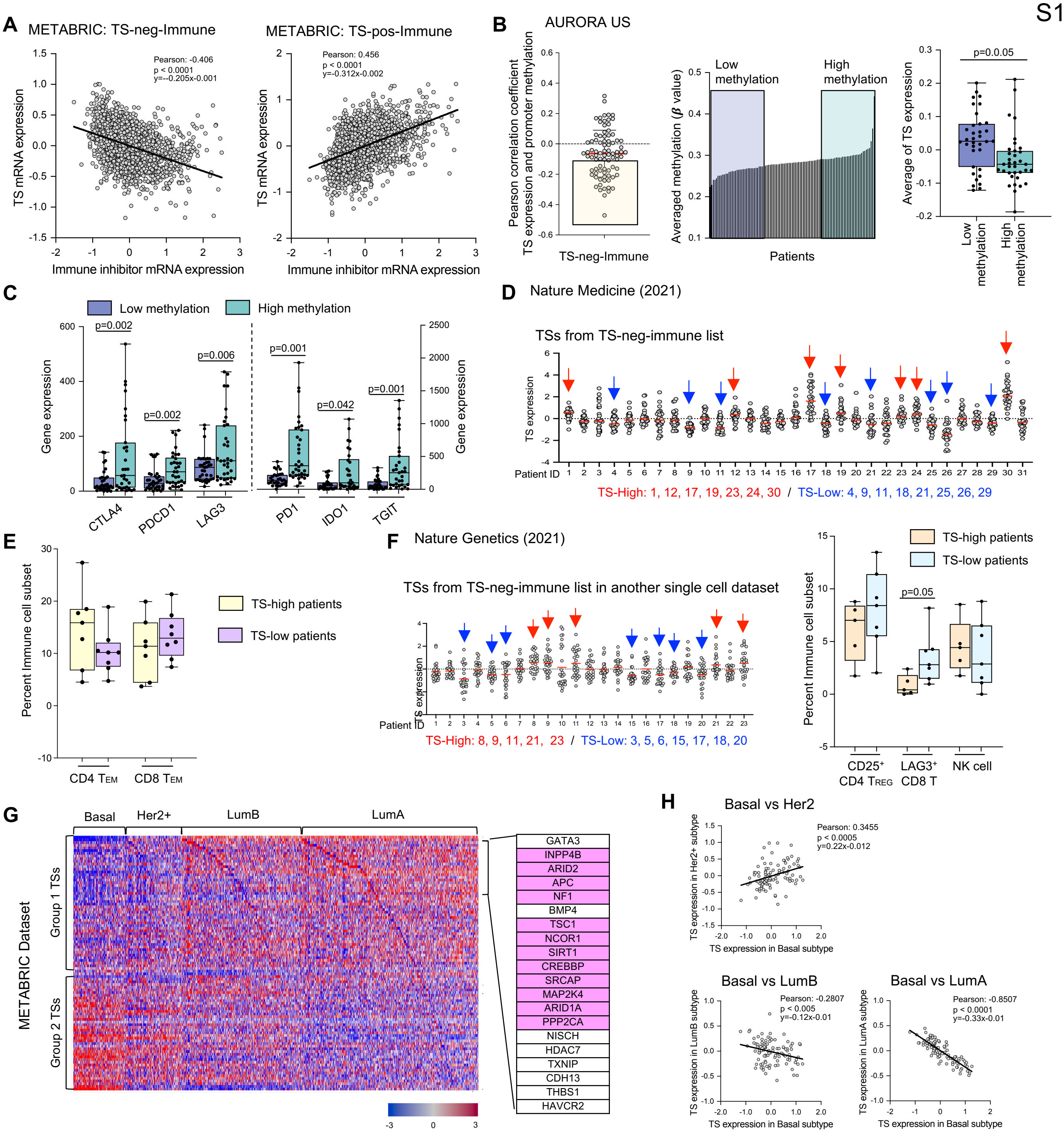

Supplementary figure 1. The association of TS expression with immune modulators and immune cell composition in breast cancer. (A) Scatter plots of mRNA expression of TS vs key Immune inhibitors from METABRIC dataset (n=1904). left panel: TS-neg-Immune from the pink boxes in Figure 1A; right panel: TS-pos-Immune from the green boxes in Figure 1A. **(B)** left panel: Pearson correlation coefficient between gene expression and promoter methylation in TS-neg-Immune genes. The genes with negative correlation (r < -0.15) were selected in yellow box; middle panel: Average promoter methylation of the selected genes: patients in low methylation (blue box) and high methylation (green box); right panel: average expression of the selected genes between low methylation (n=35) and high (n=35) methylation patients in the AURORA dataset GSE209998 and GSE212375: Garcia-Recio et al., Nature Cancer 2022. **(C)** Expression levels of immune inhibitors between low methylation (n=35) and high methylation (n=35) patients. **(D)** Expression levels of TSs from the TS-neg-Immune list in each patient with high (red arrows, n=7) or low (blue arrows, n=8). **(E)** Percentages of immune cell subsets between TS-high (n=7) and TS-low (n=8) patients in single cell dataset[28]; T_{EM}: Effector/memory T cells. **(F)** left panel: expression levels of TSs from the TS-neg-Immune list in each patient with high (red arrows, n=5) or low (blue arrows, n=7); right panel: percentage of immune cell subsets between TS-high (n=5) and TS-low (n=7) patients in another single cell dataset GSE176078 [7]: Wu et al., Nature Genetics 2021. **(G)** The expression of 102 TSs in four major subtypes of breast cancer patients in METABRIC (n=1904), right: list of top 20 Group1 TSs; pink highlight: TSs overlap with TS-neg-immune list. **(H)** Scatter plots of TS expression between different subtypes in TCGA-BRCA dataset (n=20 Group1 TSs). All box plots show the 25th percentile, the median, the 75th percentile and minimum/maximum whiskers.

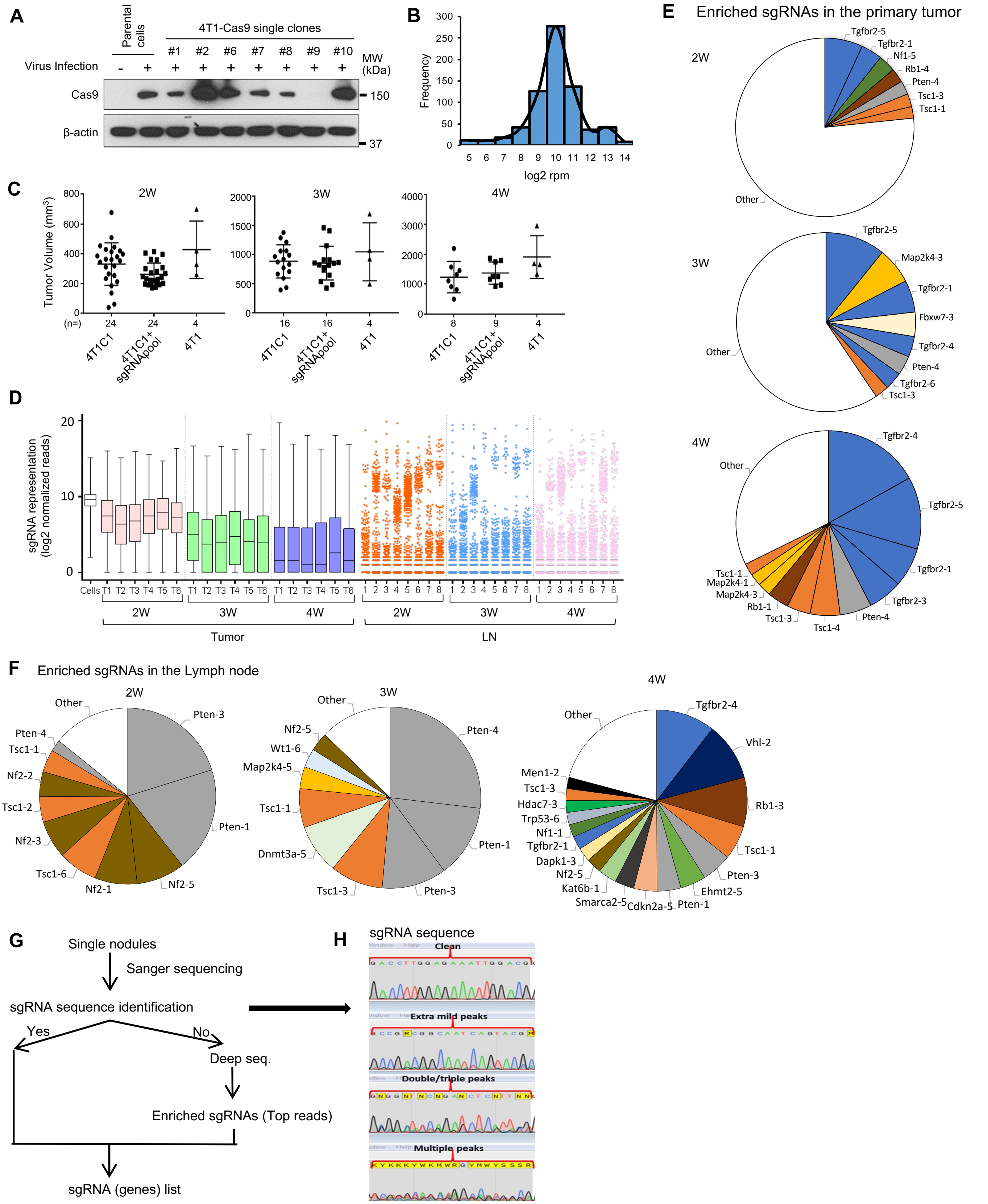

Supplementary figure 2. sgRNA screening gene candidates that suppress metastasis. (A) Cas9 Western blot from Cas9-expressing 4T1 clones. **(B)** sgRNA library representation of 4T1-C1 cells before injection. **(C)** Primary tumor growth from nude mice injected with 4T1-C1 cells and cells containing sgRNA library (n=8 mice for each group). 2 x10^5 cells were injected into mammary fat pad. Measurement of tumor size was done at 2, 3, and 4 weeks after cell injection. Graphs show mean ± s.d. **(D)** Boxplot of the sgRNA normalized read counts for injected cells, primary tumor, and lymph node during tumor progression. **(E-F)** Pie charts for the enriched sgRNAs at different stages of primary tumor **(E)** and lymph node **(F)**. **(G)** Schematic sequencing for gene identification. **(H)** Representative sanger sequencing results. Individual nodules were taken out from the lungs, PCR amplified the sgRNA sequence and examined through sanger sequencing. All sgRNAs with ≥2% of total reads are plotted individually.

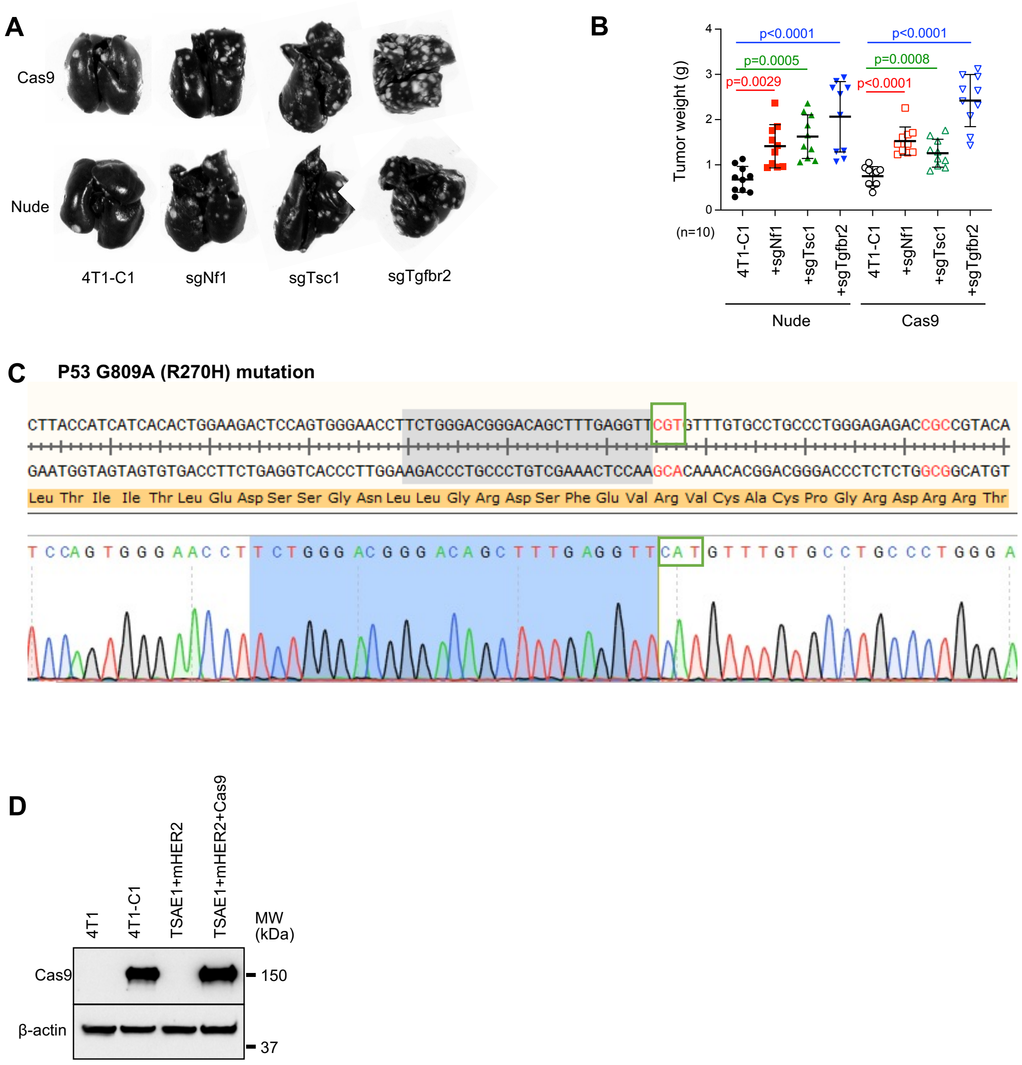

Supplementary figure 3. Deletion of genes encoding NF1, TSC1 and TβRII in nude and immune competent Cas 9 transgenic, as well as establishment of TSAE1+mHER2 cells. (A-B) representative Indian ink staining of the lungs (A) and tumor weight (B) from nude and immune competent Cas9 mice. Graph shows mean ± s.d. **(C)** Confirmation of P53 homozygous hotspot point mutation G809A (R270H) in TSAE1 cells. **(D)** Cas9 Western of TSAE1+mHER2 cells transduced with lentivirus carrying the Cas9 transgene.

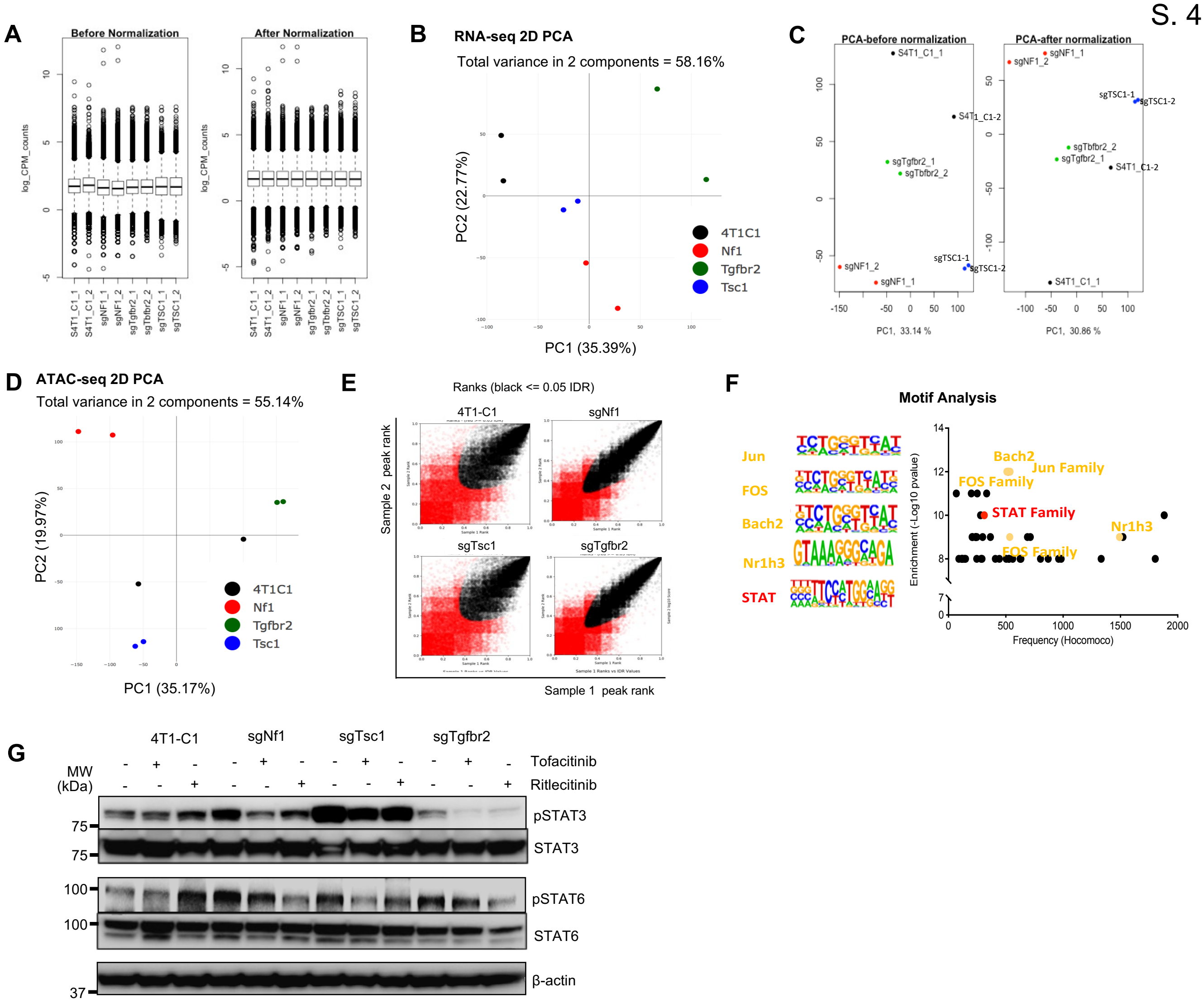

Supplementary figure 4. The tumor-cell-autonomous genes and pathways resulting from loss of TS. **(A-B)** RNAseq QC, mapped reads after normalization (A) or PCA plot (B).**(C)** PCA after normalization. **(D-E)** ATACseq PCA plot (D) and IDR value (E) of the two replicates for each sample. **(F)** Transcription motif analysis, TF enrichment (Motif match score >0.6) vs frequency (Hocomoco-Fimo (1e-5)) using Homer.**(G)** pSTAT3 and pSTAT6 Western of 4T1 cells with NF1, TSC1, and TBR2 deficiency, with C1 as control.   The cells were treated with a pan Jak inhibitor (Tofacitinib) or a Jak3 specific inhibitor (Ritlecitinib).

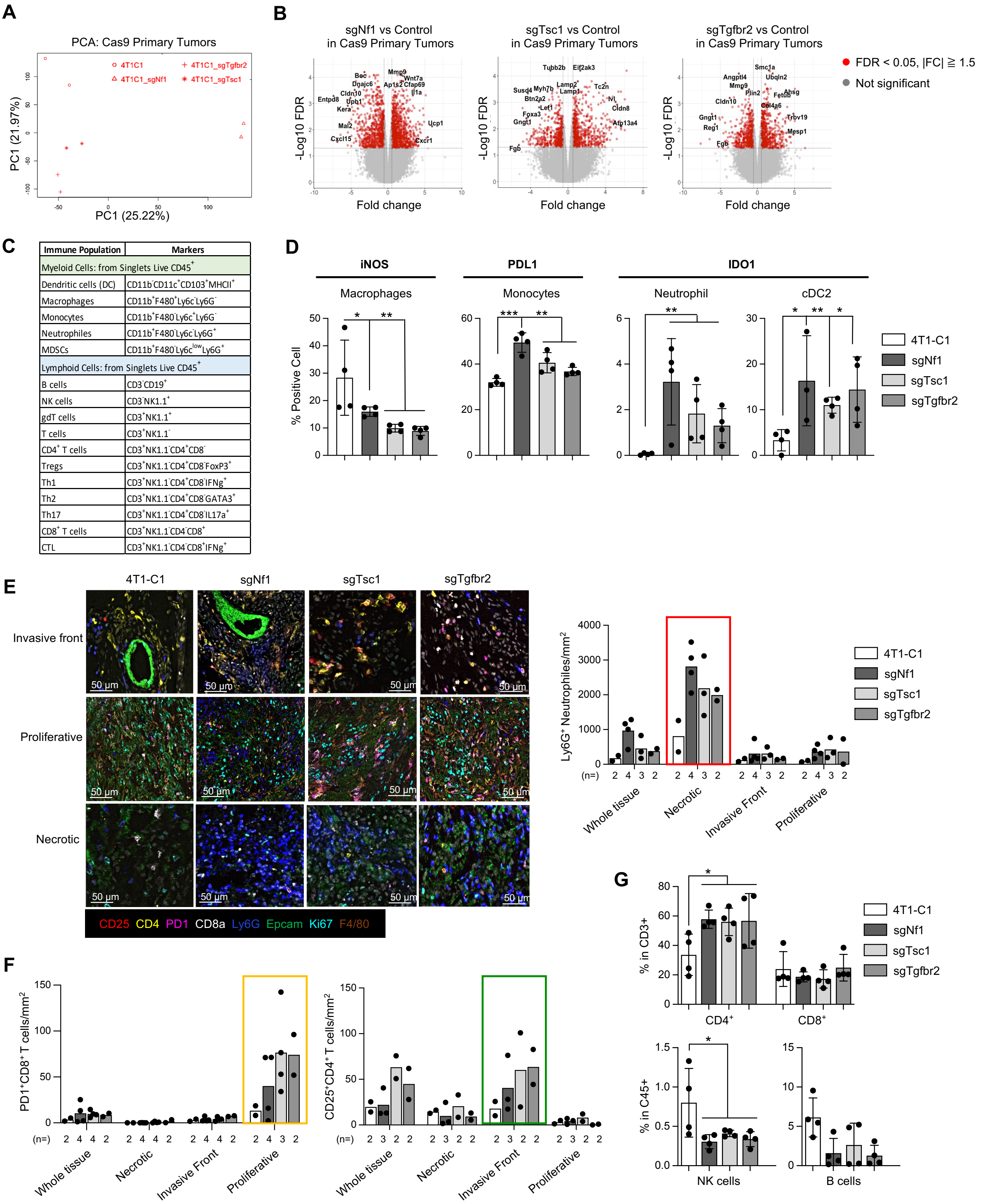

Immune Population	Markers
Myeloid Cells: from Singlets Live CD45+	
Dendritic cells (DC)	CD11b-CD11c+CD103+MHCII+
Macrophages	CD11b+F480+Ly6c-Ly6G-
Monocytes	CD11b+F480-Ly6c+Ly6G-
Neutrophiles	CD11b+F480-Ly6c-Ly6G+
MDSCs	CD11b+F480-Ly6clowLy6G+
Lymphoid Cells: from Singlets Live CD45+	
B cells	CD3-CD19+
NK cells	CD3-NK1.1+
gdT cells	CD3+NK1.1+
T cells	CD3+NK1.1-
CD4+ T cells	CD3+NK1.1-CD4+CD8-
Tregs	CD3+NK1.1-CD4+CD8-FoxP3+
Th1	CD3+NK1.1-CD4+CD8-IFNg+
Th2	CD3+NK1.1-CD4+CD8-GATA3+
Th17	CD3+NK1.1-CD4+CD8-IL17a+
CD8+ T cells	CD3+NK1.1-CD4-CD8+
CTL	CD3+NK1.1-CD4-CD8+IFNg+

Supplementary figure 5. (A) PCA plot of bulk RNASeq from primary tumor. **(B)** Differential gene expression of NF1/TSC1/TGFBR2 deficient primary tumors from cas 9 transgenic mice compared with 4T1C1 control. **(C)** list of immune markers used in CyTEK. **(D)** CyTEK analysis of myeloid cells from primary tumors with NF1, TSC1 and TβRII deficiency vs C1 control (n=4 mice per group). **(E)** Representative images from CODEX for LY6G+ neutrophils, Treg and PD1+CD8+ T cells in different regions of tumor tissues as shown, and quantitative data for LY6G+ neutrophils on the right. (**F**) Quantitative data for the PD1+CD8+ T cells and CD4+CD25+ Treg in different regions of tumor tissues. (**G**) Percentage of CD4 and CD8 T cells in CD3+ subset (upper panel), as well as percentage of NK and B cells in CD45 immune cells (n=4 mice per group). Graphs show mean ± s.d. for D and G. *, $p < 0.05$; **, $p < 0.01$; ***, $p < 0.001$.

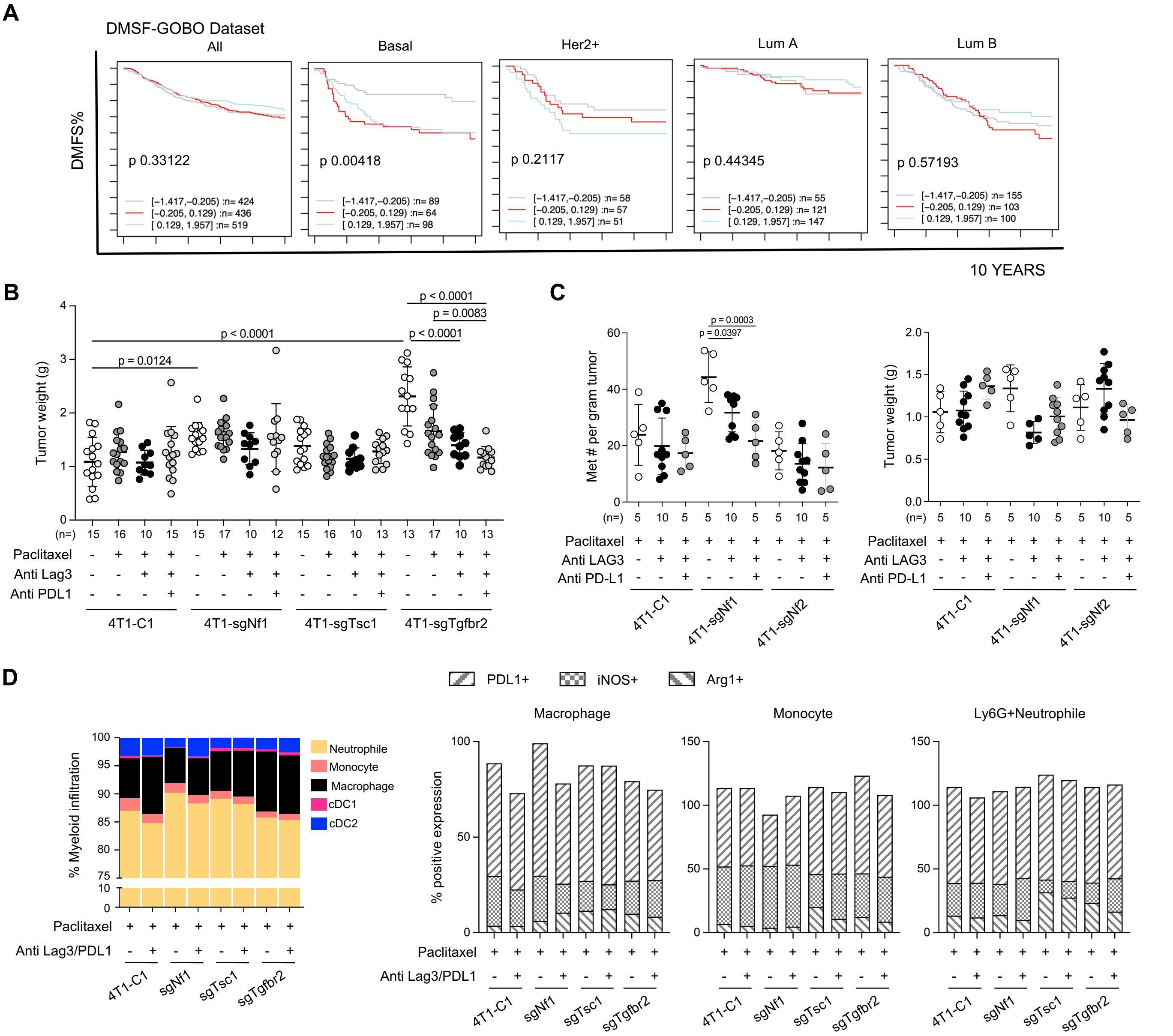

Supplementary figure 6. Loss of three tumor suppressors and immune regulation in human cancers. **(A)** significant correlations were found in TS-immune signature with DMFS of basal and Her2+ subtypes. GOBO dataset. **(B)** Tumor weight from mice that received combination treatment of anti-LAG3, anti-PD-L1 and Pac in 4T1 preclinical mouse model. **(C)** Met count normalized by tumor weight (left panel) and tumor weight (right panel) from mice that received combination treatment of anti-LAG3, anti-PD-L1 and Pac in 4T1 preclinical mouse model. **(D)** Paclitaxel (Pac), anti-LAG3 and anti-PDL1 neutralization antibodies did not change the number or the expression of PDL1, TNFa, IL6, Arg1 or iNOS in the myeloid subsets. Graph shows mean ± s.d.

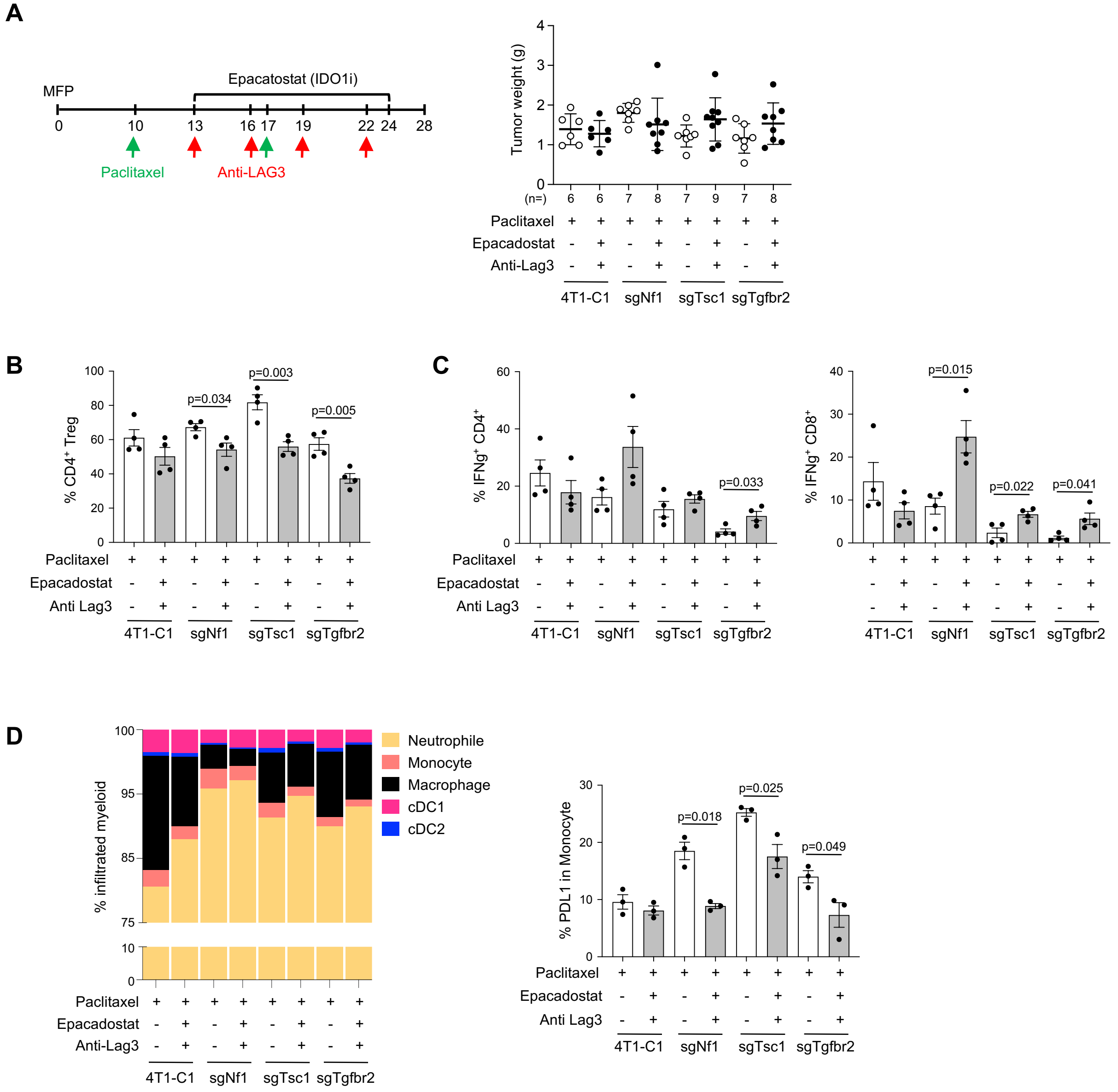

Supplementary figure 7. Targeting Lag3 and IDO1 in **4T1 preclinic mouse model bearing tumors with NF1, TSC1 and TβRII deficiency. (A)** Schematic treatment of anti-LAG3 antibody, IDO1 inhibitor and Pac, and tumor weight from treatment groups (n=6-9). Graph shows mean ± s.d. **(B-C)** CYTEK analysis showing the combination treatment decreased Treg (B, n=4 mice per group) and increased IFNg+ CD4 and CD8 T cells from treated primary tumors with NF1, TSC1 and TβRII deficiency (C, n=4 mice per group). **(D)** No difference in the number of myeloid subsets; and decreased PDL1 expression from treated primary tumors with NF1, TSC1 and TβRII deficiency (n=3 mice per group). All bar graphs show mean ± s.e.m.

Supplementary Table 1. The list of anti-mouse Antibodies using in CyTEK

Antigenes	Cat #	Clones	Company
Arg1	17-3697-82	A1exF5	eBioscience
B220	751580	RA3-6B2	BD biosciences
CD11b	612977	M1/70	BD biosciences
CD11c	117310	N418	Biolegend
CD19	561740	1D3	BD biosciences
CD206	141714	C068C2	Biolegend
CD25	102004	PC61	Biolegend
CD3	100249	17A2	Biolegend
CD4	553043	RM4-5	BD biosciences
CD40	124618	3/23	Biolegend
CD40L	751603	MR1	BD biosciences
CD44	103056	IM7	Biolegend
CD45	564279	30-F11	BD biosciences
CD62L	104410	MEL-14	Biolegend
CD80	46-0801-82	16-10A1	eBioscience
CD8a	612898	53-6.7	BD biosciences
F4/80	749283	T45-2342	BD biosciences
FoXP3	126406	MF-14	Biolegend
GATA3	565449	L50-823	BD biosciences
Granzyme B	515406	GB11	Biolegend
I-A/I-E	107608	M5/114.15.2	Biolegend
IDO1	654004	2E2/IDO1	Biolegend
IFNg	505830	XMG1.2	Biolegend
IL-10	563277	JES5-16E3	BD biosciences
IL-13	159403	W17010B	Biolegend
IL-17A	506927	TC11-18H10.1	Biolegend
IL-2	503824	JES6-5H4	Biolegend
IL-4	504118	11B11	Biolegend
IL-6	561376	MP5-20F3	BD biosciences
iNOS	12-5920-82	CXNFT	eBioscience
LAG3	125227	C9B7W	Biolegend
Ly-6G	127629	1A8	Biolegend
Ly6C	128036	HK1.4	Biolegend
NK1.1	560618	PK136	BD biosciences
PD1	109112	RMP1-30	Biolegend
PDL1	563369	MIH5	BD biosciences
Perforin	154306	S16009A	Biolegend
Tbet	561263	O4-46	BD biosciences
TCR γ/δ	118124	GL3	Biolegend
TIM3	134012	B8.2C12	Biolegend
TNFa	506338	MP6-XT22	Biolegend

Supplementary Table 2. The list of anti-mouse antibodies used in CODEX

	tag#	Target	Cat #	Clone	Commercial/custom	Dilution factor	Exposure times (msec)
1	1	CD90.2	4150001	30-H12	Akoya Biosciences	330	250
2	2	CD31	4250001	MEC13.3	Akoya Biosciences	200	250
3	3	TCRB	4550101	H57-597	Akoya Biosciences	200	450
4	5	CD44	4250002	IM7	Akoya Biosciences	400	80
5	6	PD1 (CD279)	NA	RMP1-30	Custom	110	420
6	7	CD45	4150002	30-F11	Akoya Biosciences	200	250
7	10	B220	4150006	Ra3-6B2	Akoya Biosciences	200	200
8	14	MHCII	4250003	M5	Akoya Biosciences	400	120
9	15	CD169	4550100	3D6.112	Akoya Biosciences	200	450
10	16	IgD	4150012	11-26c.2a	Akoya Biosciences	200	250
11	17	CD40	NA	3/23	Custom	100	350
12	20	CD19	4250014	6D5	Akoya Biosciences	200	350
13	21	CD3	4550109	17A2	Akoya Biosciences	200	450
14	22	CD24	4150014	M1/69	Akoya Biosciences	200	250
15	23	CD21/35	4250015	7E9	Akoya Biosciences	200	350
16	24	LY6G	4550110	1A8	Akoya Biosciences	180	400
17	25	CD11b	4150015	M1/70	Akoya Biosciences	200	250
18	26	CD4	4250016	RM4-5	Akoya Biosciences	200	350
19	27	CD71	4550111	RI7217	Akoya Biosciences	200	450
20	29	CD8A	4250017	53-6.7	Akoya Biosciences	200	350
21	30	CD11c	4550108	N418	Akoya Biosciences	200	450
22	32	F4/80	NA	T45-2342	Custom	130	280
23	33	CD49f	4550102	GoH3	Akoya Biosciences	130	450
24	35	CD25	NA	PC61	Custom	100	350
25	41	aSMA	NA	1A4	Custom	100	250
26	43	EPCAM	NA	G8.8	Custom	100	250
27	47	Ki67	4250019	B56	Akoya Biosciences	200	350

Supplementary Table 3. shRNA sequences

shRNA	Cat #	shRNA sequence	Target gene
shNf1 #1	TRCN0000034341	CCGGCCCTTCTTCTTACTGATATTTCTCGAGAAATATCAGTAAGAAGAAGGGTTTTTG	mNf1
shNf1 #2	TRCN0000034343	CCGGCCAAGCTAGAAGTGGCCTTATCTCGAGATAAGGCCACTTCTAGCTTGGTTTTTG	
shTsc1 #1	TRCN0000238187	CCGGTTGTCACATCCGTATAGTAAACTCGAGTTTACTATACGGATGTGACAATTTTTG	mTsc1
shTsc1 #2	TRCN0000238189	CCGGCAACACGTTGGTTGATTATTACTCGAGTTTACTATACGGATGTGACAATTTTTG	
shTsc1 #3	TRCN0000244252	CCGGGCAAAGCTGCTACCCGTTTATCTCGAGATAAACGGGTAGCAGCTTTGCTTTTTG	
shTgfbr2 #1	TRCN0000022626	CCGGGCTCGCTGAACACTACCAAATCTCGAGATTTGGTAGTGTTCAGCGAGCTTTTT	mTgfbr2
shTgfbr2 #2	TRCN0000022627	CCGGCCAGATCGTGTGTGAGACTTTCTCGAGAAAGTCTCACACACGATCTGGTTTTT	
shTgfbr2 #3	TRCN0000294600	CCGGGAAGGACATCTTCTCCGATATCTCGAGATATCGGAGAAGATGTCCTTCTTTTTG	

Supplementary Table 4. Primer sequence for qPCR

Primer name	Oligo sequence (5' to 3')	Targe gene
IL1_S1_FP	TCAGCACCACTTGGTTAAATGA	mIl1
IL1_S1_RP	TTAGAGTCGTCTCCTCCCGAC	
Ido1_S1_FP	AAGCATTGGAAAAGGCACTG	mIdo1
Ido1_S1_RP	GGAGAACGTGGAAAAACGTG	
Ccl2_S1_FP	TCTCTCTTCCTCCACCACCA	mCcl2
Ccl2_S1_RP	AGTGGGGCGTTAACTGCAT	
Gapdh_S1_FP	GGTGAAGGTCGGTGTGAACG	mGapdh
Gapdh_S1_RP	CTCGCTCCTGGAAGATGGTG	
Actb_S1_FP	TTACTGCTCTGGCTCCTAGCA	mActb
Actb_S1_RP	GACTCATCGTACTCCTGCTTGC	
Jak3_S1_FP	CACAGTGCATGGCCTATGAT	mJak3
Jak3_S1_RP	AGGTGTGGGGTCTGAGAGG	
