# Supplementary material for: Loss of tumor suppressors promotes inflammatory tumor microenvironment and enhances LAG3+T cell mediated immune suppression
Source: Nat Commun. 2024 Jul 12;15:5873. doi: 10.1038/s41467-024-50262-8 (PMC11245525; doi:10.1038/s41467-024-50262-8)
Supplement: Supplementary file 3 — Reporting Summary [file 41467_2024_50262_MOESM3_ESM.pdf]

nature portfolio

Corresponding author(s): Li Yang

Last updated by author(s): 2024-2-29

Reporting Summary

Nature Portfolio wishes to improve the reproducibility of the work that we publish. This form provides structure for consistency and transparency in reporting. For further information on Nature Portfolio policies, see our Editorial Policies and the Editorial Policy Checklist.

Statistics

For all statistical analyses, confirm that the following items are present in the figure legend, table legend, main text, or Methods section.

n/a	Confirmed	
☐	☒	The exact sample size (*n*) for each experimental group/condition, given as a discrete number and unit of measurement
☐	☒	A statement on whether measurements were taken from distinct samples or whether the same sample was measured repeatedly
☐	☒	The statistical test(s) used AND whether they are one- or two-sided<br>*Only common tests should be described solely by name; describe more complex techniques in the Methods section.*
☐	☒	A description of all covariates tested
☐	☒	A description of any assumptions or corrections, such as tests of normality and adjustment for multiple comparisons
☐	☒	A full description of the statistical parameters including central tendency (e.g. means) or other basic estimates (e.g. regression coefficient) AND variation (e.g. standard deviation) or associated estimates of uncertainty (e.g. confidence intervals)
☐	☒	For null hypothesis testing, the test statistic (e.g. *F*, *t*, *r*) with confidence intervals, effect sizes, degrees of freedom and *P* value noted<br>*Give P values as exact values whenever suitable.*
☒	☐	For Bayesian analysis, information on the choice of priors and Markov chain Monte Carlo settings
☒	☐	For hierarchical and complex designs, identification of the appropriate level for tests and full reporting of outcomes
☐	☒	Estimates of effect sizes (e.g. Cohen's *d*, Pearson's *r*), indicating how they were calculated

Our web collection on statistics for biologists contains articles on many of the points above.

Software and code

Policy information about availability of computer code

Data collection	All software for data collection is detailed in the manuscript. CyTEK Aurora software. Image Lab software (v 6.0.1). CODEX processor (v1.7.06). Quantstudio 6 Flex Real-Time PCR system.
Data analysis	All software for data analysis is detailed in the manuscript. Sequencing data: Cutadapt (v 1.18), STAR (v 2.5.2b), RSEM (v 1.3.0), Bowtie2 (v 2.2.6), Limma (v 3.34.9), MAcs (v 2.1.2), Deeptools (v 3.1.2). CODEFACS. GraphPad Prism 7.0 software. FlowJo analysis software (v 10.6.0). ImageJ.

For manuscripts utilizing custom algorithms or software that are central to the research but not yet described in published literature, software must be made available to editors and reviewers. We strongly encourage code deposition in a community repository (e.g. GitHub). See the Nature Portfolio guidelines for submitting code & software for further information.

Data

Policy information about availability of data

All manuscripts must include a data availability statement. This statement should provide the following information, where applicable:

- Accession codes, unique identifiers, or web links for publicly available datasets
- A description of any restrictions on data availability
- For clinical datasets or third party data, please ensure that the statement adheres to our policy

The data supporting the findings of this study are available within the article and the supplementary information files are available from the corresponding author

upon request. The raw and processed sequencing data from ATAC-seq and RNA-seq have been deposited in the GEO database and are publicly available under accession numbers GSE249967 (https://www.ncbi.nlm.nih.gov/geo/query/acc.cgi?acc=GSE249967), GSE249969 https://www.ncbi.nlm.nih.gov/geo/query/acc.cgi?acc=GSE249969) and GSE249971 (https://www.ncbi.nlm.nih.gov/geo/query/acc.cgi?acc=GSE249971). All relevant source data for each figure are provided.

Human research participants

Policy information about studies involving human research participants and Sex and Gender in Research.

Reporting on sex and gender	N/A
Population characteristics	N/A
Recruitment	N/A
Ethics oversight	N/A

Note that full information on the approval of the study protocol must also be provided in the manuscript.

Field-specific reporting

Please select the one below that is the best fit for your research. If you are not sure, read the appropriate sections before making your selection.

☒ Life sciences ☐ Behavioural & social sciences ☐ Ecological, evolutionary & environmental sciences

For a reference copy of the document with all sections, see nature.com/documents/nr-reporting-summary-flat.pdf

Life sciences study design

All studies must disclose on these points even when the disclosure is negative.

Sample size	The sample sizes were based on the previous publications (e.g., Cancer Res. 80(12): 2612-2627, 2020; Mol. Carcnimogenesis, Jul; 59(7):679-690, 2020) and on accepted standards in the field.
Data exclusions	No data were excluded from the analyses, except for Figure 5F, left panel, due to a noted technical problem.
Replication	All in vitro results were obtained from at least two repeating biological experiment with success . The in vivo experiments were performed with animal numbers that give statistical significance, and the animal numbers were provided in all in vivo study.
Randomization	All mice in vivo study were randomized before cancer cell injection and drug treatment, and in vivo samples were randomly selected for downstream experiments.
Blinding	Immune profiling using CyTEK were performed blindly by separate researchers. Phenotype of animals experiments was not evaluated blindly, and major results were verified by separate researchers.

Reporting for specific materials, systems and methods

We require information from authors about some types of materials, experimental systems and methods used in many studies. Here, indicate whether each material, system or method listed is relevant to your study. If you are not sure if a list item applies to your research, read the appropriate section before selecting a response.

Materials & experimental systems

n/a	Involved in the study	
☐	☒	Antibodies
☐	☒	Eukaryotic cell lines
☒	☐	Palaeontology and archaeology
☐	☒	Animals and other organisms
☒	☐	Clinical data
☒	☐	Dual use research of concern

Methods

n/a	Involved in the study	
☒	☐	ChIP-seq
☐	☒	Flow cytometry
☒	☐	MRI-based neuroimaging

Antibodies

Antibodies used	For Western Blot: Primary antibodies, Cas9 (Diagenode, C15200203, 7A9), NF1 (abcam, ab17963), TSC1 (ThermoFisher, PA5-20131),

Antibodies used	TGF-betaR2 (R&D System, AF532), NF2 (Cell Signaling, #6995, D1D8), Pten (Cell Signaling, #9559, 138G6), Caspase 3 (Cell Signaling, #9665, 8G10), pAKT (Cell Signaling, #9271), AKT (Cell Signaling, #9272), pERK (Cell Signaling, #9101), ERK (Santa Cruz, sc-514302, C-9), pmTOR (Santa Cruz, sc-293133, 59.Ser2448), HER2 (Cell Signaling, #2165, 29D8), CD40 (R&D System, AF440) , pSTAT3 (Cell Signaling, #9145S, D3A7), STAT3 (Cell Signaling, #4904S, 79D7), pSTAT6 (Cell Signaling, #56554S, D8S9Y and 9361S), STAT6 (Cell Signaling, #9362S), pJAK3 (Cell Signaling, #5031S, D44E3), JAK3 (Cell Signaling, #8863S, D7B12), or β-actin (Santa Cruz, sc-69879, AC-15). Secondary antibodies, HRP-conjugated anti-mouse (Cell Signaling, #7076S), anti-rabbit (Cell Signaling, #7074S) and anti-goat (Invitrogen, #31400).<br>For Immunofluorescence or immunohitochemical staining: Priamry antibodies, E-Cadherin (BD Transduction Laboratories™, BD610181, C36), E-Cadherin (Cell Signaling, #3195, 24E10), NF1 (1:200, abcam, ab17963), TSC1 (ThermoFisher, PA5-20131), TGF-betaRII (R&D System, MAB532, C129502). Secondary antibodies, goat anti-mouse Alexa Fluor 594 (A11005, Invitrogen), donkey anti-rabbit Alexa Fluor 488 (A21206, Invitrogen), donkey anti-rabbit Alexa Fluor 594 (A21207, Invitrogen), or donkey anti-rat Alexa Fluor 488 (A21208, Invitrogen).<br>For CyTEK: Arg1 (eBioscience, 17-3697-82, A1exF5), B220 (BD biosciences, 751580, RA3-6B2), CD11b (BD biosciences, 612977, M1/70), CD11c (Biolegend, 117310, N418), CD19 (BD biosciences, 561740, 1D3), CD206 (Biolegend, 141714, C068C2), CD25 (Biolegend, 102004, PC61), CD3 (Biolegend, 100249, 17A2), CD4 (BD biosciences, 553043, RM4-5), CD40 (Biolegend, 124618, 45374), CD40L (BD biosciences, 751603, MR1), CD44 (Biolegend, 103056, IM7), CD45 (BD biosciences, 564279, 30-F11), CD62L (Biolegend, 104410, MEL-14), CD80 (eBioscience, 46-0801-82, 16-10A1), CD8a (BD biosciences, 612898, 53-6.7), F4/80 (BD biosciences, 749283, T45-2342), FoXP3 (Biolegend, 126406, MF-14), GATA3 (BD biosciences, 565449, L50-823), Granzyme B (Biolegend, 515406, GB11), I-A/I-E (Biolegend, 107608, M5/114.15.2), IDO1 (Biolegend, 654004, 2E2/IDO1), IFNg (Biolegend, 505830, XMG1.2), IL-10 (BD biosciences, 563277, JES5-16E3), IL-13 (Biolegend, 159403, W17010B), IL-17A (Biolegend, 506927, TC11-18H10.1), IL-2 (Biolegend, 503824, JES6-5H4), IL-4 (Biolegend, 504118, 11B11), IL-6 (BD biosciences, 561376, MP5-20F3), iNOS (eBioscience, 12-5920-82, CXNFT), LAG3 (Biolegend, 125227, C9B7W), Ly-6G (Biolegend, 127629, 1A8), Ly6C (Biolegend, 128036, HK1.4), NK1.1 (BD biosciences, 560618, PK136), PD1 (Biolegend, 109112, RMP1-30), PDL1 (BD biosciences, 563369, MIH5), Perforin (Biolegend, 154306, S16009A), Tbet (BD biosciences, 561263, O4-46), TCR γ/δ (Biolegend, 118124, GL3), TIM3 (Biolegend, 134012, B8.2C12), TNFa (Biolegend, 506338, MP6-XT22).<br>For CODEX: CD90.2 (Akoya Biosciences, 4150001, 30-H12), CD31 (Akoya Biosciences, 4250001, MEC13.3), TCRB (Akoya Biosciences, 4550101, H57-597), CD44 (Akoya Biosciences, 4250002, IM7), CD45 (Akoya Biosciences, 4150002, 30-F11), B220 (Akoya Biosciences, 4150006, Ra3-6B2), MHCII (Akoya Biosciences, 4250003, M5), CD169 (Akoya Biosciences, 4550100, 3D6.112), IgD (Akoya Biosciences, 4150012, 11-26c.2a), CD19 (Akoya Biosciences, 4250014, 6D5), CD3 (Akoya Biosciences, 4550109, 17A2), CD24 (Akoya Biosciences, 4150014, M1/69), CD21/35 (Akoya Biosciences, 4250015, 7000000000), LY6G (Akoya Biosciences, 4550110, 1A8), CD11b (Akoya Biosciences, 4150015, M1/70), CD4 (Akoya Biosciences, 4250016, RM4-5), CD71 (Akoya Biosciences, 4550111, RI7217), CD8A (Akoya Biosciences, 4250017, 53-6.7), CD11c (Akoya Biosciences, 4550108, N418), CD49f (Akoya Biosciences, 4550102, GoH3), Ki67 (Akoya Biosciences, 4250019, B56).
Validation	All antiboides were vaildated for mouse and for given application on the manufacturer's websites, unless stated below.<br>E-cadherin (BD610181): J Cell Biol. 2001; 155(4):531-542.<br>NF1 (ab17963): Molecules 2023, 28(13), 5128.<br>TGF-betaRII (MAB532): No literature available, validated in comparions to IgG control staining.<br>The costom-made CODEX antibodies, PD1 (CD279) (RMP1-30), CD40 (3/23), F4/80 (T45-2342), CD25 (PC61), aSMA (1A4), and EPCAM (G8.8), were validated the Collaborative Protein Technology Resource Core at NCI.

Eukaryotic cell lines

Policy information about cell lines and Sex and Gender in Research

Cell line source(s)	Murine 4T1, EMT6 cell lines: ATCC.<br>TSAE1 cell line were gifted from Lalage M. Wakefield.
Authentication	Authentication has not been performed.
Mycoplasma contamination	All cell lines were mycoplasma negative.
Commonly misidentified lines (See ICLAC register)	Non of commonly misidentified cell lines were used in the study.

Animals and other research organisms

Policy information about studies involving animals; ARRIVE guidelines recommended for reporting animal research, and Sex and Gender in Research

Laboratory animals	Mice, BALB/c, female, 6-8 week old.<br>Mice, athymic nu/nu, female, 6-8 week old.<br>Mice, BALB/c-Gt(ROSA)26Sortm1(CAG-cas9*,-EGFP)Fezh, female, 6-8 week old.
Wild animals	No wild animals were used in the study.
Reporting on sex	Female mice were only used for the study because of our focus on breast cancer.
Field-collected samples	No field-collected samples were used in the study.
Ethics oversight	All animal protocols were approved by National Cancer Institute's Animal Care and Use Committee.

Note that full information on the approval of the study protocol must also be provided in the manuscript.

Flow Cytometry

Plots

Confirm that:

☒ The axis labels state the marker and fluorochrome used (e.g. CD4-FITC).

☒ The axis scales are clearly visible. Include numbers along axes only for bottom left plot of group (a 'group' is an analysis of identical markers).

☒ All plots are contour plots with outliers or pseudocolor plots.

☒ A numerical value for number of cells or percentage (with statistics) is provided.

Methodology

Sample preparation	The primary tumors were minced and incubated with tissue dissociation buffer (RPMI containing 1mg/ml Collagenase, 0.012g/ml of Dispase and 0.015 g/ml of DNase I) for 40-45 minutes with rotation (150 rpm at 37C). Dissociated tissues were then filtered by 70 um cell strainer and washed with MACS buffer (PBS containing 2 % FBS and 1 mM EDTA). Red blood cells were removed by incubation with ACK buffer. After washing with PBS buffer, cells were stained with LIVE/DEAD fixable Blue dye. Cells were stained with appropriate antibodies before and after fixation. Detailed lists of antibodies were provided in the manuscript.
Instrument	CyTEK Aurora.
Software	CyTEK Aurora software and FlowJo software (v 10.6.0).
Cell population abundance	Minimum 5 million cells per sample were analyzed by CyTEK. 15 ~ 20 % cells were live/CD45+ cell population. The abundance of subset cell populations were determined in percentage from parental populations.
Gating strategy	Debris removal: FSC-A/SSC-A, Single cell selection: FSC-A/FSC-H and SSC-A/SSC-H, Live CD45+ cells: CD45/LiveDead dye. From the live CD45+ cells, two separate gating strategies were utilized for myeloid and lymphoid cells. Myeloid cells; Macrophages: F4_80+/CD11b+, Neutrophile: Ly6G-high/Ly6C+, Monocytes: Ly6G+/Ly6C-high, cDC1: CD11b-/CD11c+, cDC2: CD11b+/CD11c+. Lymphoid cells; NK cells: NK1.1+/CD3-, CD4 T cells: CD3+/CD4+/CD8-, CD8 T cells: CD3+/CD8+/CD4-, B cells: CD3-/CD19+.

☒ Tick this box to confirm that a figure exemplifying the gating strategy is provided in the Supplementary Information.
