# Supplementary material for: Loss of tumor suppressors promotes inflammatory tumor microenvironment and enhances LAG3+T cell mediated immune suppression
Source: Nat Commun. 2024 Jul 12;15:5873. doi: 10.1038/s41467-024-50262-8 (PMC11245525; doi:10.1038/s41467-024-50262-8)
Supplement: Supplementary file 4 — Source Data [file 41467_2024_50262_MOESM4_ESM.zip › Raw_WB.pdf]

Fig. 3A- Western Blot of knock out of individual candidate TSs sgNf1/Tsc1/Tgfbr2/Nf2

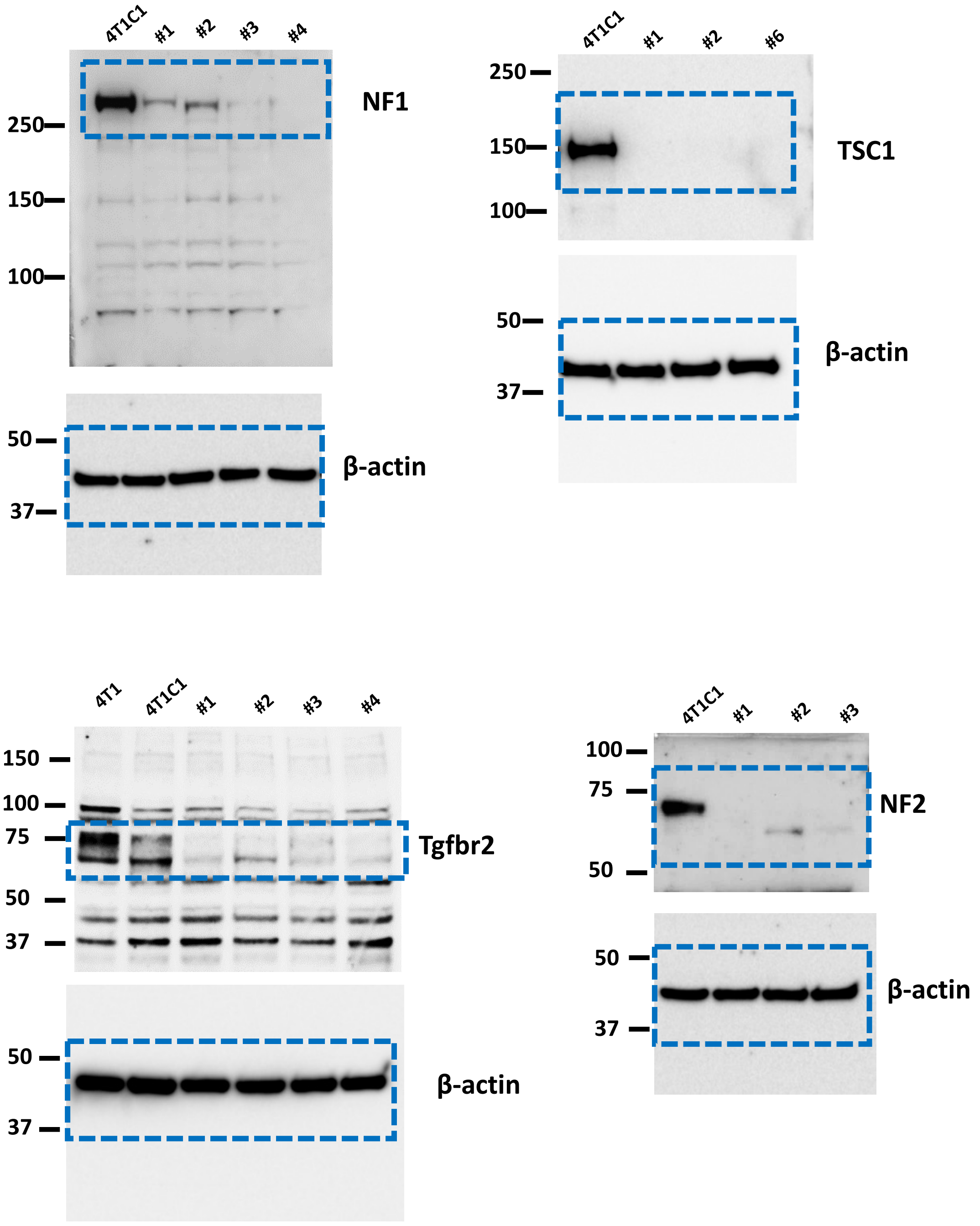

Fig. 3G - Western Blot of overexpression of HER2 in TSAE1 cell line

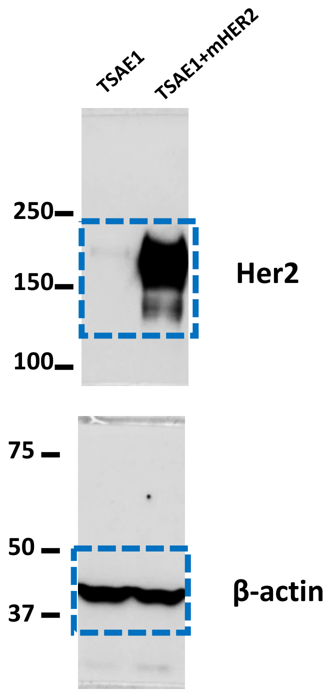

Fig. 3H- Western Blot of knock out of individual candidate TSs sgNf1/Tsc1/Tgfbr2/Nf2 in TSAE1-Her2+ cell line

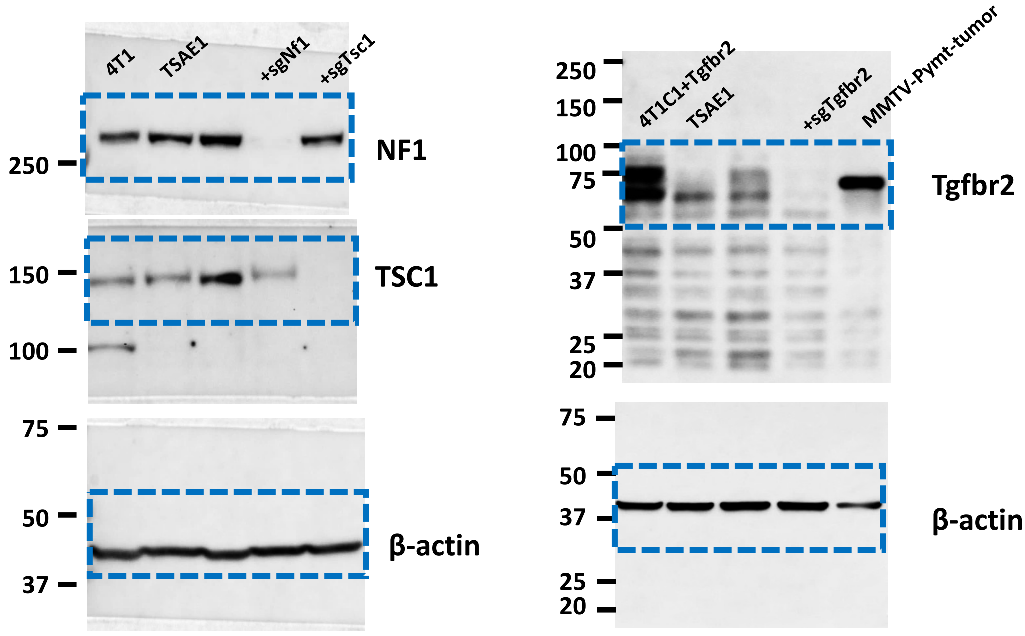

Fig. 4A: 4T1 Mouse Model

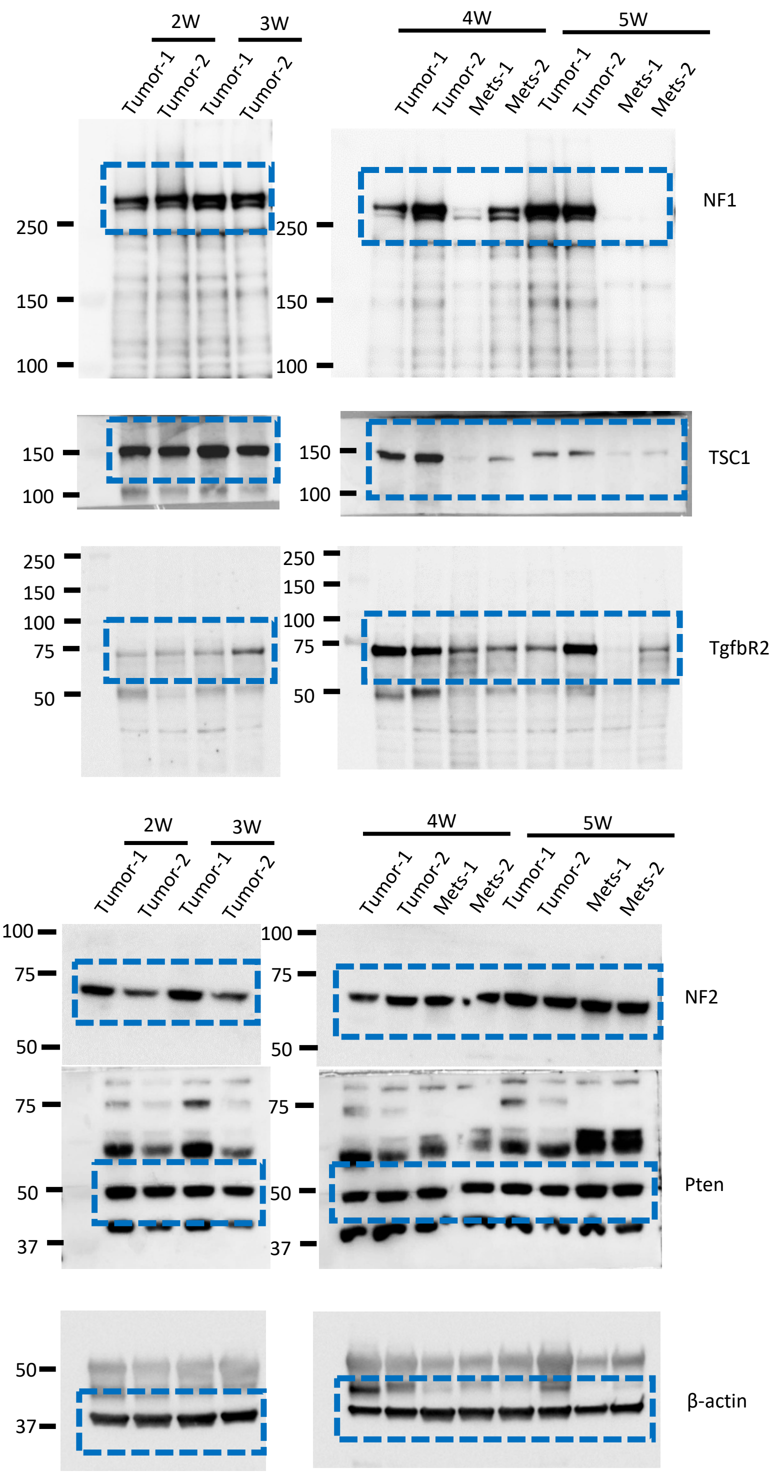

Fig. 4B: EMT6 Mouse Model

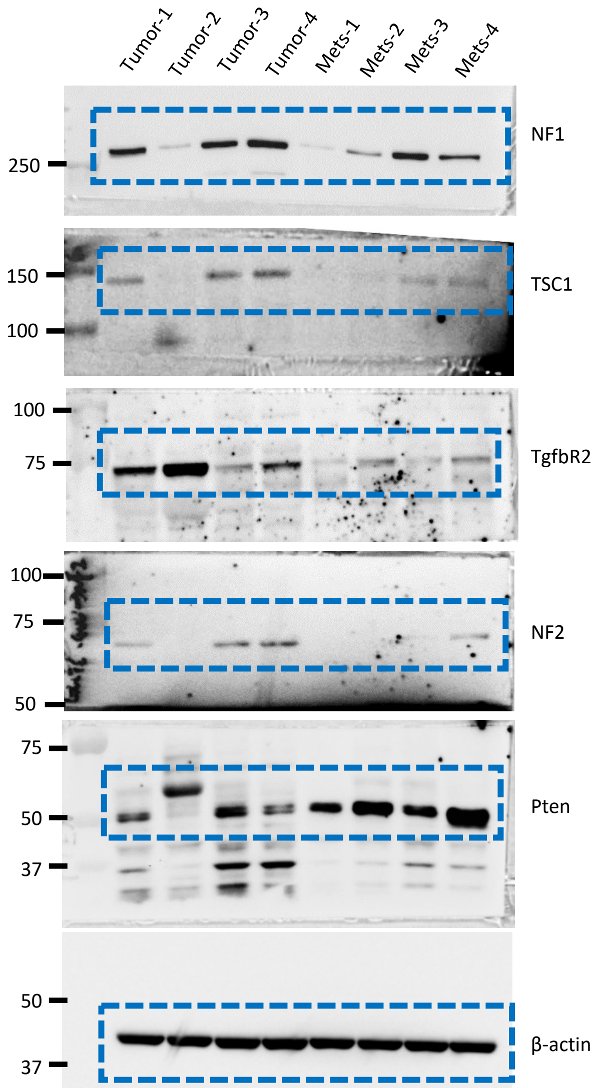

Fig. 5F- Western Blot of Jak3 expressed in 4T1C1 and +sgNf1/Tsc1/Tgfbr2

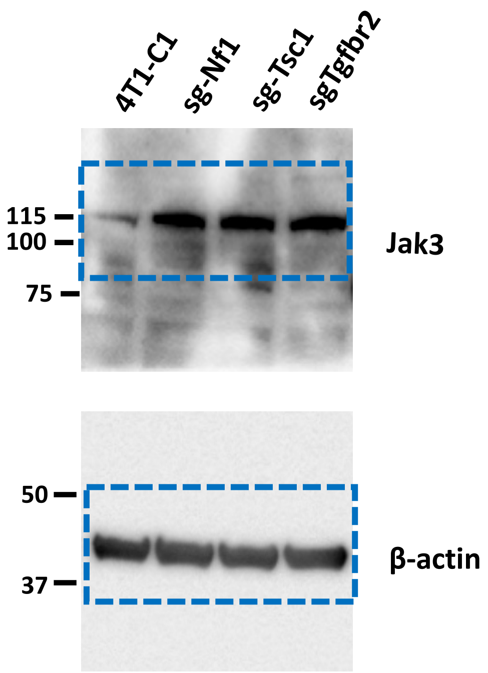

Fig. 5G- Western Blot of phosphorylation of Stat3/6 and Jak3 in 4T1C1 and +sgNf1/Tsc1/Tgfbr2

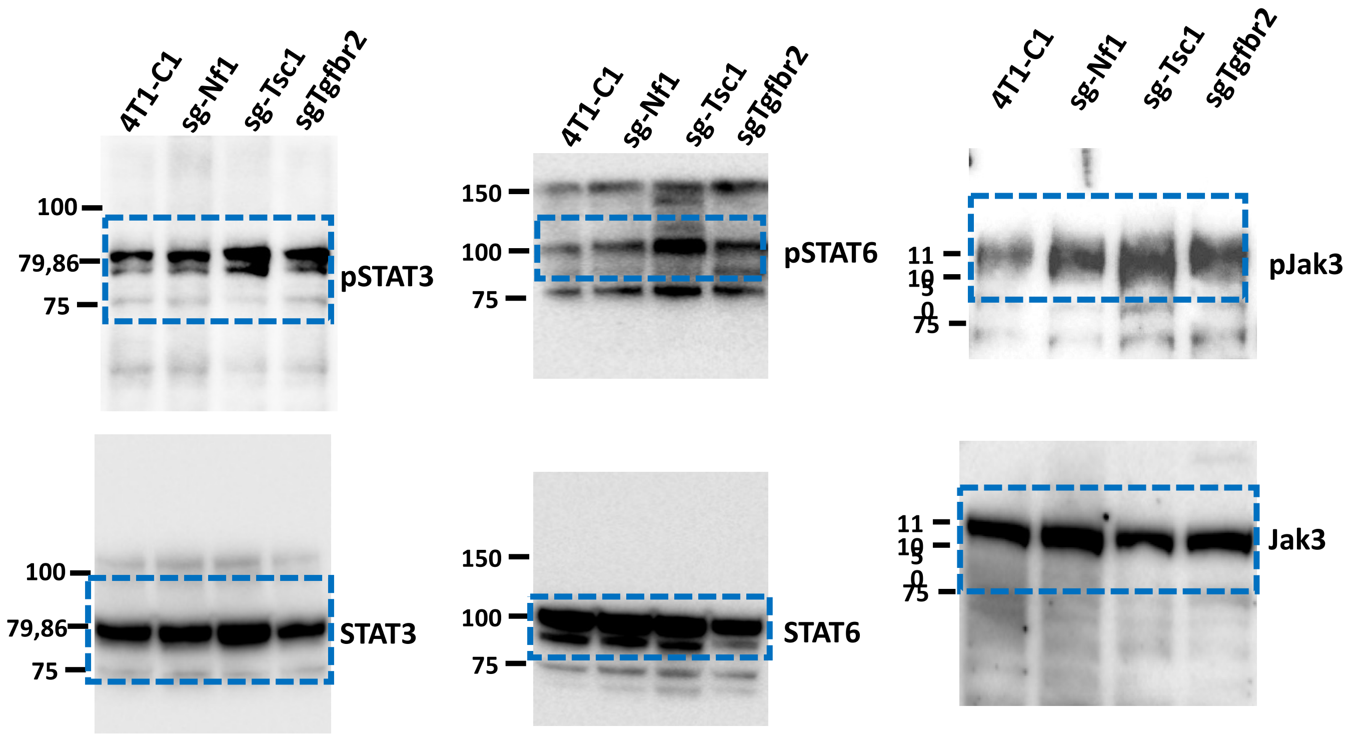

Fig. S2A

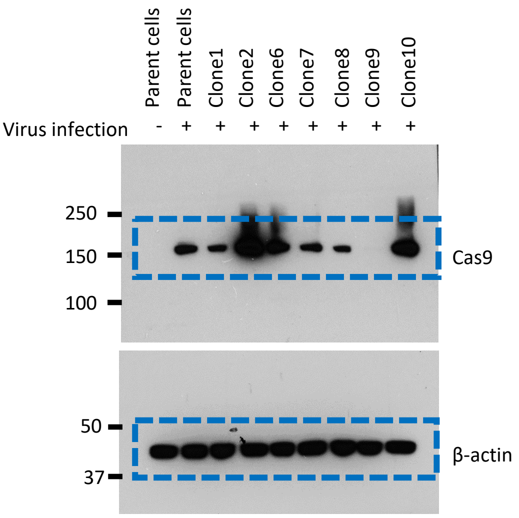

Fig. S3D

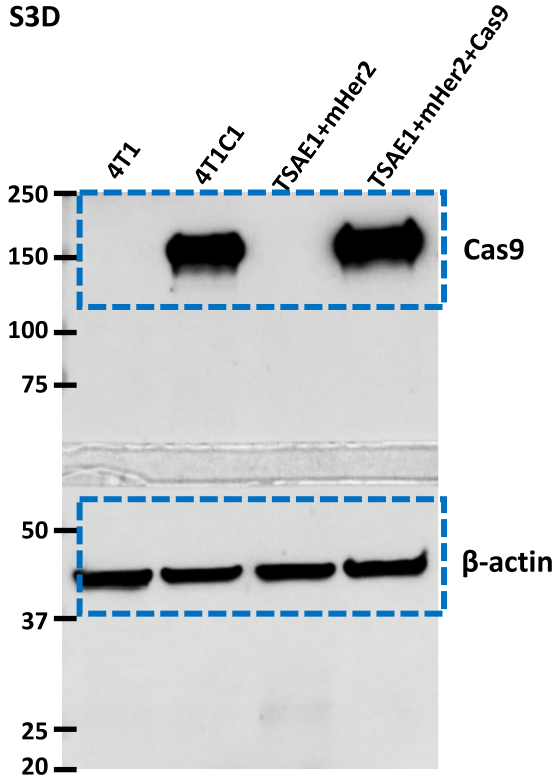

Fig. S4G

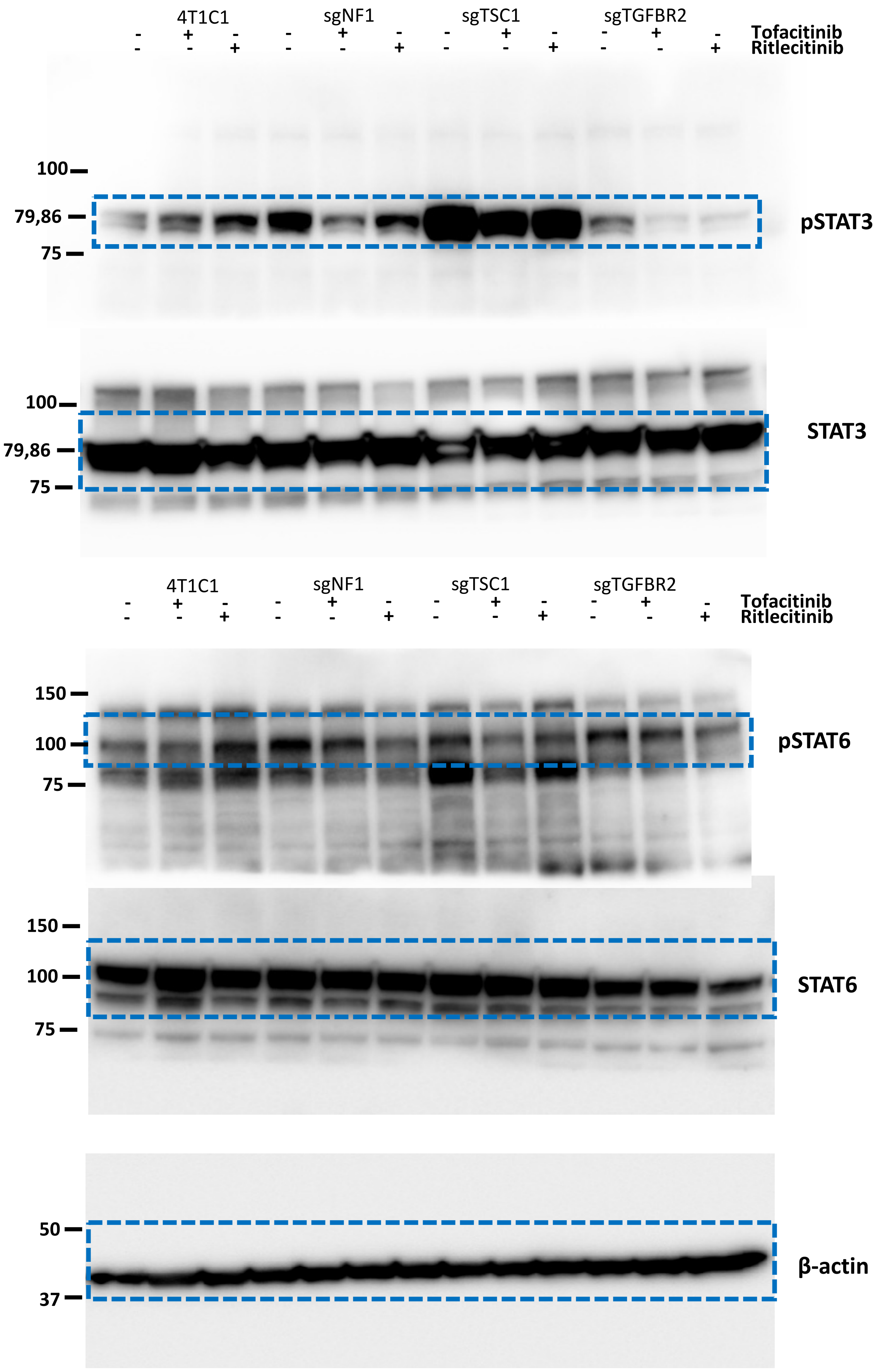
4T1C1
sgNF1
sgTSC1
sgTGFBR2
Tofacitinib
Ritlecitinib
100
79,86
75
pSTAT3
100
79,86
75
STAT3
4T1C1
sgNF1
sgTSC1
sgTGFBR2
Tofacitinib
Ritlecitinib
150
100
75
pSTAT6
150
100
75
STAT6
50
37
β-actin
